# Complete Genome Sequence of Thiostrepton-Producing *Streptomyces laurentii* ATCC 31255

**DOI:** 10.1128/genomeA.00360-16

**Published:** 2016-06-02

**Authors:** Katsumi Doi, Yasuhiro Fujino, Yuko Nagayoshi, Toshihisa Ohshima, Seiya Ogata

**Affiliations:** a Microbial Genetic Division, Institute of Genetic Resources, Faculty of Agriculture, Kyushu University, Fukuoka, Japan; b Division for Arts and Science, Faculty of Arts and Science, Kyushu University, Fukuoka, Japan; c Department of Biomedical Engineering, Faculty of Engineering, Osaka Institute of Technology, Osaka, Japan

## Abstract

*Streptomyces laurentii* ATCC 31255 produces thiostrepton, a thiopeptide class antibiotic. Here, we report the complete genome sequence for this strain, which contains a total of 8,032,664 bp, 7,452 predicted coding sequences, and a G+C content of 72.3%.

## GENOME ANNOUNCEMENT

*Streptomyces* species are Gram-positive aerobic mycelial bacteria belonging to the family *Streptomycetaceae* (1). These bacteria have the ability to produce a wide variety of secondary metabolites, including antibiotics (2), and are also interesting subjects with which to study morphological differentiation (3). Pock formation involving conjugative plasmids is a typical physiological characteristic used for morphological differentiation of *Streptomyces* species (4), which contain both plasmids and a large linear chromosome.

*Streptomyces laurentii* ATCC 31255 was classified as a thiostrepton producer not involving *Streptomyces azureus*, *Streptomyces hawaiiensis*, or *Streptomyces* sp. strain X-14b (5). It is distinct from the *Streptomyces fradiae* group (5), and *Streptomyces termitum* and *Streptomyces roseofulvus* are contained within the *S. laurentii* group (1). The thiostrepton resistance gene (*tsr*) is a selective marker often used in the genetic engineering of actinomycetes and was isolated from the strain (6). We previously reported that *S. laurentii* contains a linear conjugative plasmid, pSLL (7), and circular integrative plasmid, pSLS (8). pSLL suppressed the injurious effects caused by pSLS, which include marked decreases in spore formation and thiostrepton productivity. The whole-genome sequence of *S. laurentii* may shed light on the mechanisms of spore formation and thiostrepton production and may also be useful for comparative studies of morphological and metabolic differentiation among thiostrepton-producing *Streptomyces* species.

A sample was prepared for sequencing by growing *S. laurentii* ATCC 31255 aerobically overnight at 28°C in tryptic soy broth (TSB) (Oxoid). The genomic DNA was then extracted and purified, as we described previously (9). The prepared genome was sequenced using the PacBio RSII platform; 150,292 raw reads resulted in 100,282 quality-filtered trimmed reads, yielding 741 Mb, with a mean genome-wide coverage of 77.43×. The filtered reads were assembled using HGAP version 2.3.0 (10) and resulted in a 1-contig scaffold. Annotation was performed using Microbial Genome Annotation Pipeline (MiGAP; http://www.migap.org/).

The genome of *S. laurentii* ATCC 31255 includes a linear chromosome, with a G+C content of 72.3%. Annotation using the COG, RefSeq, and TrEMBL databases with tRNAscan-SE version 1.23 and additional manual inspection revealed 7,452 predicted coding regions, 69 tRNA genes, and 7 rRNA genes.

Terminal inverted repeats are contained at the end of the chromosome of *S. laurentii*. In addition, a gene cluster for thiostrepton biosynthesis was annotated from genes SLA3859 (*tsrS*) to SLA3877 (*tsrA*) (11). This cluster shows strong similarity to the thiostrepton biosynthesis cluster of *S. azureus* (12). The *tsr* gene is located between the EF-Tu gene and the *N*-acetylmuramoyl-l-alanine amidase family protein-coding gene. One copy of the pSLS sequence is integrated between SLA2930 and SLA2952, and a second copy is between SLA3776 and SLA3796. Plasmid pSLL does not exist in free form within its host cell.

### Nucleotide sequence accession number.

The *S. laurentii* ATCC 31255 genome sequence and annotation data have been deposited in the DDBJ/EMBL/GenBank under accession no. AP017424.
